# Early Sensory Dysfunction in Type 2 Diabetes: Vibration Perception Abnormalities in Patients Without Neuropathic Symptoms

**DOI:** 10.3390/biomedicines14102289

**Published:** 2026-10-09

**Authors:** Saule Abaykyzy Altynbekova, Yelmurat Sharipuly Omar, Tolganay Kaldykhanovna Serikkanova, Zhangentkhan Abylaiuly, Mavlyuda Bakhromkizi Ibraimova, Svetlana Viktorovna Bolshakova

**Affiliations:** Department of Endocrinology, Asfendiyarov Kazakh National Medical University, Almaty 050012, Kazakhstan; saule_90_9@mail.ru (S.A.A.);

**Keywords:** type 2 diabetes mellitus, diabetic peripheral neuropathy, vibration perception threshold, biothesiometry, peripheral sensory dysfunction, sensory assessment

## Abstract

**Background/Objectives:** Type 2 diabetes mellitus (T2DM) is associated with peripheral sensory dysfunction, and sensory abnormalities may develop before the onset of clinically apparent neuropathic symptoms. This study aimed to compare vibration perception thresholds (VPTs) between patients with T2DM without self-reported symptoms of peripheral neuropathy and individuals without disorders of glucose metabolism and to determine whether T2DM is independently associated with higher VPT. **Methods:** This cross-sectional study included 160 participants: 98 patients with T2DM and 62 controls. VPT was assessed using the Biothesi-VPT device at eight standardized anatomical sites on each foot. Group comparisons were performed using the Mann–Whitney U, Kruskal–Wallis, and Pearson’s chi-square tests. Multivariable linear regression was used to assess the association between T2DM and overall VPT after adjustment for age, sex, and body mass index (BMI). **Results:** Patients with T2DM had significantly higher VPTs at all anatomical sites on both feet (all *p* < 0.001). Median integrated VPT was 16.5 V (IQR, 10.2–25.2) versus 5.6 V (IQR, 2.8–11.1) for the right foot and 16.2 V (IQR, 9.7–25.6) versus 6.6 V (IQR, 2.6–10.5) for the left foot in patients with T2DM and controls, respectively (both *p* = 0.001). After adjustment for age, sex, and BMI, T2DM remained independently associated with higher overall VPT (B = 6.482; 95% CI, 3.495–9.477; *p* < 0.001). Age was also independently associated with higher VPT, whereas sex and BMI were not. ABI did not differ significantly between groups. **Conclusions:** Patients with T2DM without self-reported neuropathic symptoms had impaired vibration perception compared with controls, and T2DM remained associated with higher VPT after adjustment for age, sex, and BMI. Quantitative VPT assessment may help identify early peripheral sensory dysfunction in T2DM, although its diagnostic and prognostic value requires prospective evaluation.

## 1. Introduction

Diabetes mellitus remains one of the major medical and public health challenges worldwide due to the high prevalence of chronic complications that substantially impair patients’ quality of life and increase the risk of disability [1]. Among its neurological complications, peripheral nervous system involvement is of particular importance, as it develops progressively and may remain unrecognized for a prolonged period [2]. The pathogenesis of diabetic peripheral neuropathy (DPN) is multifactorial and involves chronic hyperglycemia, nerve ischemia, oxidative stress, inflammation, and metabolic disturbances [3,4].

DPN develops in a substantial proportion of patients during the course of diabetes and is characterized by progressive peripheral nerve damage resulting in sensory, motor, and autonomic dysfunction. Its clinical significance is largely attributable to the increased risk of diabetic foot complications, chronic neuropathic pain, and mortality [5]. Despite advances in diabetes management, the early recognition of DPN remains challenging. According to the American Diabetes Association, up to 50% of cases of diabetic peripheral neuropathy may be asymptomatic, potentially delaying recognition and increasing the risk of subsequent complications [6].

In patients with type 2 diabetes mellitus (T2DM), peripheral sensory abnormalities may already be present at the time of diabetes diagnosis, as hyperglycemia can remain undetected for a prolonged period. The reported prevalence of peripheral neuropathy among adults with diabetes ranges from 6% to 51%, with clinical presentations varying from asymptomatic disease to painful neuropathy [7]. Diabetic neuropathy is also associated with a substantial risk of foot ulceration and lower-extremity amputation, highlighting the importance of active screening and timely identification [7]. The prevalence of DPN increases with diabetes duration: it has been reported in approximately 26% of patients five years after disease onset and in 41% after ten years, reflecting the progressive nature of peripheral nerve involvement [8].

Conventional clinical neurological assessment may have limited sensitivity for detecting early sensory abnormalities, particularly when such abnormalities are not accompanied by typical signs or symptoms on routine examination [9].

Electrophysiological methods, including nerve conduction studies, provide objective assessment of peripheral nerve dysfunction; however, they predominantly evaluate large myelinated nerve fibers and may not adequately detect early small-fiber involvement [10]. Morphological assessment of small nerve fibers can be achieved using invasive techniques, such as skin or nerve biopsy; however, their routine clinical application is limited by invasiveness, technical complexity, and limited accessibility [11].

Quantitative assessment of vibration perception represents a practical approach to the evaluation of sensory dysfunction. Biothesiometry enables quantitative measurement of the vibration perception threshold (VPT) and may therefore facilitate the identification of sensory abnormalities that are not apparent on routine clinical assessment [12,13].

VPT primarily reflects the functional integrity of large myelinated sensory fibers and can be quantitatively assessed using devices such as Biothesi-VPT, Vibrasense, and the Neurothesiometer. VPT measurements have demonstrated associations with neurophysiological parameters and clinically relevant outcomes, while the reproducibility of VPT testing has also been evaluated in population-based studies [12,13].

This study aimed to compare VPTs between patients with T2DM without self-reported neuropathic symptoms and individuals without disorders of glucose metabolism and to assess the independent association between T2DM and higher VPTs.

## 2. Materials and Methods

### 2.1. Study Design and Participants

A cross-sectional study was conducted to compare vibration perception thresholds between patients with type 2 diabetes mellitus without self-reported symptoms of peripheral neuropathy and individuals without known disorders of glucose metabolism.

The study was conducted at the Center for Internal Medicine of Asfendiyarov Kazakh National Medical University, Almaty, Kazakhstan, between June and August 2026. A total of 160 participants were included: 98 patients with previously diagnosed T2DM and 62 control participants without known disorders of glucose metabolism. A previous diagnosis of T2DM in the study group was based on participants’ self-reported medical history. Control participants were classified as individuals who reported no history of diabetes or other known disorders of glucose metabolism. At the time of enrollment, none of the participants reported clinical symptoms suggestive of peripheral neuropathy.

The study protocol was approved by the Local Ethics Committee of Asfendiyarov Kazakh National Medical University (Protocol No. 4 (178), dated 29 May 2026). Written informed consent was obtained from all participants prior to enrollment. The study was conducted in accordance with the principles of the Declaration of Helsinki.

### 2.2. Inclusion and Exclusion Criteria

The inclusion criteria were as follows: age ≥18 years; a self-reported previous diagnosis of T2DM for the study group; no known disorders of glucose metabolism, as reported by the participant, for the control group; and no self-reported symptoms suggestive of peripheral neuropathy at the time of enrollment.

The exclusion criteria were type 1 diabetes mellitus or other specific types of diabetes; known peripheral neuropathy of other etiologies; severe foot deformities; acute inflammatory conditions or foot ulcers; severe decompensated systemic disease; previous lower-limb amputation; and any condition that precluded vibration perception testing.

### 2.3. Assessment of Vibration Perception Threshold

Vibration perception threshold was assessed using a Biothesi-VPT biothesiometer (KODYS, Chennai, India). Measurements were performed in the morning under stable room-temperature conditions.

The intensity of the vibration stimulus was gradually increased from 0 V until the participant first reported perceiving the vibration. The stimulus intensity at the point of first perception was recorded as the VPT (V). A higher VPT indicated lower vibration sensitivity.

VPT was assessed at eight anatomical sites on each foot: the great toe; first metatarsal head (1st MTH); third metatarsal head (3rd MTH); fifth metatarsal head (5th MTH); medial aspect of the midfoot; heel; central aspect of the dorsum of the foot; and medial malleolus.

Measurements were performed by physicians according to a standardized study protocol. Each participant was assessed entirely by the same physician. At each anatomical site, VPT was measured three times, and the arithmetic mean of the three consecutive measurements was used for analysis.

For an overall assessment of vibration perception in each foot, an integrated VPT value was calculated as the arithmetic mean of the VPT values obtained at the eight anatomical sites.

For descriptive interpretation, VPT values <15 V were considered to indicate preserved vibration perception, values of 15–24.9 V were considered to indicate an elevated vibration perception threshold, and values ≥25 V were considered to indicate a markedly elevated vibration perception threshold [14]. This categorization was used for descriptive purposes and was not considered a standalone diagnostic criterion for diabetic peripheral neuropathy.

### 2.4. Anthropometric and Clinical Parameters

Age and sex were recorded for all participants. Body weight and height were measured, and body mass index (BMI) was calculated as body weight in kilograms divided by height in meters squared (kg/m^2^). Duration of diabetes was additionally recorded for participants with T2DM.

The ankle–brachial index (ABI) was determined separately for the right and left lower limbs in all participants. Systolic blood pressure was measured in both upper limbs and at both ankles using a sphygmomanometer and a pneumatic cuff. In each lower limb, systolic pressure was determined over the dorsalis pedis and posterior tibial arteries, with arterial pulsation assessed by palpation. After the cuff was inflated until the palpable pulse disappeared, cuff pressure was gradually reduced, and the pressure at which the pulse reappeared was recorded as the systolic blood pressure.

For each lower limb, the higher of the two systolic pressure values obtained from the dorsalis pedis and posterior tibial arteries was used to calculate the ABI. The higher systolic blood pressure measured in the right or left brachial artery was used as the denominator for both lower limbs. The ABI for each lower limb was calculated as the selected ankle systolic pressure divided by the higher brachial systolic pressure.

### 2.5. Statistical Analysis

Statistical analyses were performed using IBM SPSS Statistics, version 19.0 (IBM Corp., Armonk, NY, USA).

The distribution of quantitative variables was assessed using the Shapiro–Wilk and Kolmogorov–Smirnov tests. Quantitative variables that did not follow a normal distribution were presented as medians and interquartile ranges (median [IQR]), and comparisons between two independent groups were performed using the Mann–Whitney U test.

ABI values did not show a statistically significant deviation from a normal distribution and were therefore presented as mean ± standard deviation (mean ± SD). Differences in ABI between the two independent groups were assessed using the independent-samples Student’s *t*-test.

When VPT values were compared between the T2DM and control groups separately across the eight anatomical sites of the right and left feet, 16 comparisons were performed. The *p*-values reported are unadjusted. To account for multiple comparisons, a Bonferroni-corrected significance threshold of *p* < 0.0031 (0.05/16) was applied.

The Kruskal–Wallis test was used to compare quantitative variables across three age groups (18–44, 45–59, and ≥60 years). When a statistically significant overall difference was identified, post hoc pairwise comparisons were performed using the Mann–Whitney U test with Bonferroni correction. For the three pairwise comparisons, the adjusted significance threshold was *p* < 0.0167.

Categorical variables were presented as absolute and relative frequencies, *n* (%). Between-group differences in categorical variables were assessed using Pearson’s χ^2^ test; Fisher’s exact test was used when the assumptions of the χ^2^ test were not met.

For multivariable analysis, an overall VPT value was calculated as the mean of the integrated VPT values of the right and left feet. Multivariable linear regression analysis was performed with overall VPT as the dependent variable and T2DM status, age, sex, and BMI as independent variables. Regression assumptions were assessed by visual inspection of residual-versus-fitted plots for linearity and homoscedasticity, histograms and normal P–P plots of standardized residuals for residual normality, and Cook’s distance for influential observations. Heteroscedasticity was additionally assessed using an auxiliary regression of squared unstandardized residuals on the model predictors. Because evidence of heteroscedasticity was detected, HC3 heteroskedasticity-consistent robust standard errors were used for coefficient inference. Unstandardized regression coefficients (B), HC3 robust standard errors (SE), standardized coefficients (β), 95% confidence intervals (CIs), and *p*-values were reported. The coefficient of determination (R^2^) and adjusted R^2^ were used to describe the overall model.

For all other analyses not subject to adjustment for multiple comparisons, a two-sided *p*-value <0.05 was considered statistically significant.

## 3. Results

### 3.1. Characteristics of the Study Population

A total of 160 participants were included in the study, of whom 98 (61.2%) patients with T2DM comprised the study group, and 62 (38.8%) individuals without known disorders of glucose metabolism comprised the control group. The overall study population included 97 (60.6%) women and 63 (39.4%) men. The sex distribution did not differ significantly between the groups (*p* = 0.072).

The median age of the overall study population was 58.5 years (IQR 46.2–65.0). Importantly, a substantial baseline age imbalance was observed between the groups, with participants with T2DM being markedly older than those in the control group (median age, 61.0 years [IQR 54.0–67.0] vs. 43.5 years [IQR 25.7–59.2], respectively; *p* < 0.001). This marked age difference represents an important baseline imbalance and should be considered when interpreting between-group comparisons.

Participants were categorized into three age groups: 18–44 years, 45–59 years, and ≥60 years, accounting for 24.4%, 31.9%, and 43.7% of the overall study population, respectively. The proportion of participants with T2DM differed across the age groups, increasing from 17.9% among those aged 18–44 years to 70.6% among those aged 45–59 years and 78.6% among those aged ≥60 years (*p* < 0.001).

BMI also differed between the groups. The median BMI was 28.9 kg/m^2^ (IQR 24.9–32.1) in participants with T2DM compared with 26.9 kg/m^2^ (IQR 21.6–31.1) in the control group (*p* = 0.012).

Among participants with T2DM, the duration of diabetes was ≤5 years in 42 (42.9%) participants and ≥6 years in 56 (57.1%). The main characteristics of the study groups are summarized in Table 1.

### 3.2. Vibration Perception Thresholds in the Study Groups

Vibration perception threshold values were significantly higher in participants with T2DM than in the control group at all eight anatomical sites of both feet (all unadjusted *p* < 0.001). All between-group differences remained statistically significant when the Bonferroni-corrected significance threshold of *p* < 0.0031 was applied for 16 multiple comparisons.

The highest median VPT values among participants with T2DM were observed at the ankle, reaching 21.3 V (IQR 12.6–28.6) on the right and 19.2 V (IQR 11.4–27.4) on the left. The corresponding values in the control group were 6.5 V (IQR 3.2–14.0) and 6.3 V (IQR 2.9–13.5), respectively.

Between-group differences were characterized by moderate-to-large effect sizes (r = 0.48–0.56), indicating substantial differences in VPT distributions between participants with T2DM and controls. Detailed results are presented in Table 2.

The integrated vibration perception threshold, calculated as the mean value across all measurement sites on each foot, was also significantly higher in patients with T2DM than in the control group. On the right side, the median integrated VPT was 16.5 V (IQR, 10.2–25.2) in patients with T2DM compared with 5.6 V (IQR, 2.8–11.1) in controls (*p* = 0.001). On the left side, the corresponding values were 16.2 V (IQR, 9.7–25.6) and 6.6 V (IQR, 2.6–10.5), respectively (*p* = 0.001). 

Regarding diabetes duration, no statistically significant differences in integrated VPT were observed between patients with different durations of T2DM (*p* = 0.097). In patients with a T2DM duration of ≤5 years, the median VPT was 13.6 V (IQR, 8.9–26.6) for the right foot and 14.1 V (IQR, 8.5–25.6) for the left foot, compared with 18.2 V (IQR, 12.2–24.4) and 18.0 V (IQR, 13.0–25.3), respectively, in patients with a disease duration of ≥6 years.

When VPT was analyzed across age subgroups (18–44, 45–59, and ≥60 years), no statistically significant differences were observed for the right foot among patients with T2DM (*p* = 0.052), whereas the overall Kruskal–Wallis test showed a significant difference for the left foot (*p* = 0.019). However, after Bonferroni correction, none of the pairwise comparisons between age subgroups remained statistically significant (adjusted significance threshold, *p* < 0.0167). In the control group, VPT increased with age for both the right and left feet (*p* < 0.001 for both). After Bonferroni correction, significant differences remained between the 18–44 and 45–59 year groups and between the 18–44 and ≥60 year groups for both feet (*p* ≤ 0.001). Between the 45–59 and ≥60 year groups, a statistically significant difference was observed only for the right foot (*p* = 0.010). 

In the sex-stratified analysis, among patients with T2DM, the median integrated VPT of the right foot was higher in women than in men (18.9 vs. 14.5 V; *p* = 0.047), whereas no statistically significant difference was observed for the left foot (17.6 vs. 14.2 V; *p* = 0.205). In the control group, VPT did not differ significantly between women and men for either the right (*p* = 0.117) or left foot (*p* = 0.268).

Multivariable linear regression analysis showed that T2DM remained independently associated with a higher overall VPT after adjustment for age, sex, and BMI (B = 6.482; HC3 95% CI, 3.495–9.477; β = 0.308; *p* < 0.001). Age was also independently associated with a higher VPT (B = 0.263; HC3 95% CI, 0.162–0.363; β = 0.400; *p* < 0.001), whereas neither sex (B = 0.271; HC3 95% CI, −2.588 to 3.133; *p* = 0.851) nor BMI (B = −0.049; HC3 95% CI, −0.343 to 0.246; *p* = 0.743) showed a statistically significant independent association with VPT. The model explained 36.7% of the variance in overall VPT (R^2^ = 0.367; adjusted R^2^ = 0.351). Given evidence of heteroscedasticity in the regression diagnostics, HC3 heteroscedasticity-consistent robust standard errors were used for coefficient inference (Table 3).

No statistically significant differences in the ankle–brachial index were observed between the study groups. The mean right-sided ABI was 1.13 (SD 0.11) in patients with T2DM and 1.15 (SD 0.14) in the control group (*p* = 0.069), while the corresponding left-sided values were 1.13 (SD 0.11) and 1.16 (SD 0.12), respectively (*p* = 0.083). No statistically significant differences in ABI were observed according to T2DM duration. In patients with a T2DM duration of ≤5 years, the mean ABI values were 1.151 (SD 0.123) on the right and 1.157 (SD 0.129) on the left, compared with 1.124 (SD 0.105) and 1.121 (SD 0.109), respectively, in patients with a disease duration of ≥6 years (right: *p* = 0.238; left: *p* = 0.140).

## 4. Discussion

In the present study, patients with type 2 diabetes mellitus had significantly higher vibration perception threshold values than individuals without disorders of glucose metabolism. These differences were observed at all anatomical sites assessed on both feet and were also evident for the integrated VPT measures. Our findings are consistent with previous evidence demonstrating a high prevalence of peripheral sensory dysfunction in T2DM and support the potential utility of quantitative vibration perception assessment for detecting sensory impairment [6,15].

The higher VPT values observed in patients with T2DM are consistent with previous studies in which quantitative assessment of vibration perception was used to identify peripheral sensory dysfunction and diabetic polyneuropathy. Liu et al. demonstrated the diagnostic utility of VPT in the assessment of diabetic polyneuropathy, while Hu et al. reported a strong association between VPT and the severity of peripheral neuropathy [16,17]. In the present study, median integrated VPT values were substantially higher in patients with T2DM than in the control group. These findings indicate impaired vibration perception in patients with T2DM in the absence of self-reported neuropathic symptoms and are consistent with evidence that sensory abnormalities may occur before clinically apparent peripheral neuropathy [18].

Age is an important factor influencing vibration perception. In the control group, VPT progressively increased with age in both feet, consistent with previously reported age-related declines in vibration sensitivity [19]. Among patients with T2DM, age-related differences were less pronounced: the overall Kruskal–Wallis test identified differences for the left foot, but no statistically significant pairwise differences between age categories remained after Bonferroni correction. Nevertheless, multivariable regression analysis confirmed that age was independently associated with a higher overall VPT (B = 0.263; HC3 95% CI, 0.162–0.363; *p* < 0.001).

Importantly, T2DM remained statistically associated with higher VPT after adjustment for age, sex, and BMI; however, substantial residual confounding by age cannot be excluded (B = 6.482; HC3 95% CI, 3.495–9.477; β = 0.308; *p* < 0.001). Therefore, the adjusted association between T2DM and higher VPT should be interpreted with caution, as the observed between-group differences may be partly attributable to the age-related decline in vibration sensitivity [19]. 

When VPT was analyzed according to diabetes duration, median values were numerically higher in patients with a T2DM duration of ≥6 years than in those with a disease duration of ≤5 years; however, the difference did not reach statistical significance (*p* = 0.097). Previous studies have demonstrated changes in VPT and other components of sensorimotor function in relation to T2DM duration, including among patients without clinically established peripheral neuropathy [20]. The absence of a statistically significant difference in the present study may be related to subgroup size, variability in VPT values, and the influence of other clinical and metabolic factors. Therefore, these findings should be regarded as descriptive and require confirmation in larger studies.

In the sex-stratified analysis, women with T2DM had a higher VPT in the right foot than men (*p* = 0.047), whereas no statistically significant difference was observed for the left foot. Previous studies have shown that VPT may be influenced by demographic and anthropometric factors, including age, height, and, depending on the anatomical site of measurement, sex [21]. In the present study, however, sex was not independently associated with overall VPT after adjustment for T2DM, age, and BMI (B = 0.271; 95% CI, −2.525 to 3.067; *p* = 0.849). Accordingly, the unadjusted difference observed for the right foot should be interpreted with caution.

Vibration perception predominantly reflects the function of large myelinated sensory fibers, impairment of which is a component of diabetic polyneuropathy [6,12]. Chronic hyperglycemia, oxidative stress, microvascular dysfunction, and other metabolic mechanisms are recognized contributors to peripheral nerve injury in T2DM [22]. Therefore, an elevated VPT may reflect dysfunction of large sensory fibers even in the absence of subjective neuropathic symptoms. Nevertheless, quantitative VPT assessment alone is insufficient to establish a diagnosis of diabetic peripheral neuropathy and should be considered a quantitative measure of sensory function alongside clinical and, when indicated, electrophysiological assessments.

An additional finding of the present study was the absence of statistically significant differences in the ankle–brachial index between patients with T2DM and the control group. Despite the marked between-group differences in VPT, mean ABI values were comparable for both lower extremities. This indicates that the differences in vibration perception observed in our study were not accompanied by corresponding between-group differences in ABI. However, the absence of differences in ABI does not completely exclude a contribution of vascular abnormalities, as this measure does not capture all aspects of peripheral circulation.

The present study has several limitations. The cross-sectional design precludes establishing a causal relationship between T2DM and elevated VPT and does not allow assessment of changes in sensory function over time. A further important limitation is the substantial age imbalance between the study groups, with participants with T2DM being considerably older than controls and with limited overlap in the age distributions. Because vibration perception declines with age, this imbalance represents an important potential source of confounding. Although multivariable regression was used to adjust for age, statistical adjustment cannot fully compensate for the limited representation of younger participants with T2DM and older participants in the control group. Therefore, residual confounding by age is likely, and the independent association between T2DM and higher VPT should be interpreted with caution. Future studies using age-matched control groups are needed to confirm these findings.

Another limitation concerns the reliance on self-reported information for group classification and the assessment of neuropathic symptoms. A previous diagnosis of T2DM, the absence of known disorders of glucose metabolism, and the absence of neuropathic symptoms were determined based on participants’ self-reports and were not systematically confirmed by laboratory or comprehensive neurological assessment. This may have introduced a risk of misclassification, particularly with respect to unrecognized disturbances of glucose metabolism in the control group and unrecognized neuropathic conditions. In addition, data on glycemic control, including HbA1c, were not available for all participants with T2DM, limiting a comprehensive assessment of their glycemic status and precluding a reliable evaluation of the association between glycemic control and VPT. Furthermore, although participants with known peripheral neuropathy of other etiologies and severe decompensated systemic disease were excluded, smoking, alcohol use, renal function, vitamin B12 status, and other potential confounding factors were not systematically assessed or included in the multivariable model; therefore, residual confounding from these factors cannot be excluded. Finally, quantitative VPT assessment was not accompanied by electrophysiological evaluation of peripheral nerve function; therefore, the findings primarily reflect impairment of vibration perception and should not be interpreted as independent confirmation of diabetic peripheral neuropathy. 

The strengths of this study include quantitative VPT assessment at multiple standardized anatomical sites on both feet, the inclusion of a control group, and the use of multivariable analysis to account for the potential effects of age, sex, and BMI. The detection of elevated VPT in patients with T2DM without self-reported neuropathic symptoms supports further investigation of quantitative vibration perception assessment as a potential tool for the early detection of peripheral sensory dysfunction.

Future studies should include longitudinal follow-up with repeated VPT measurements to characterize changes in sensory function over time and identify factors associated with their progression. Prospective evidence indicates that VPT may change during long-term follow-up in patients with diabetes, although the course of progression varies considerably among individuals [23]. Further studies should also compare VPT with clinical neuropathy scores, nerve conduction studies, and measures of metabolic control, including HbA1c. Larger multicenter studies with age-matched control groups are warranted to more precisely determine the independent association between T2DM and impaired vibration perception and to establish the clinical utility of VPT for the early detection of peripheral sensory dysfunction.

## 5. Conclusions

Patients with type 2 diabetes mellitus without self-reported neuropathic symptoms had significantly higher vibration perception threshold values than individuals without known disorders of glucose metabolism at all anatomical sites assessed on both feet. These findings indicate impaired vibration perception in the absence of self-reported neuropathic symptoms.

After adjustment for age, sex, and body mass index, T2DM remained independently associated with a higher VPT. Age was also independently associated with increased VPT, whereas no independent associations were observed for sex or BMI. Quantitative VPT assessment may be useful for the early detection of peripheral sensory dysfunction in patients with T2DM; however, its diagnostic and prognostic value requires further evaluation in prospective studies.

## Figures and Tables

**Table 1:** Demographic, clinical, and anthropometric characteristics of the study participants.

Characteristic	Overall (*n* = 160)	T2DM (*n* = 98)	Control Group (*n* = 62)	*p*-Value
Sex, *n* (%)				0.072
Male	63 (39.4)	44 (44.9)	19 (30.6)	
Female	97 (60.6)	54 (55.1)	43 (69.4)	
Age, years, median (IQR)	58.5 (46.2–65.0)	61.0 (54.0–67.0)	43.5 (25.7–59.2)	<0.001
BMI, kg/m^2^, median (IQR)	28.1 (24.2–31.7)	28.9 (24.9–32.1)	26.9 (21.6–31.1)	0.012
Diabetes duration, *n* (%)				
≤5 years	-	42 (42.9)	-	-
≥6 years	-	56 (57.1)	-	-

Data are presented as median (IQR) for continuous variables and *n* (%) for categorical variables. Between-group comparisons of continuous variables were performed using the Mann–Whitney U test. Sex distribution was compared using Pearson’s χ2 test. Diabetes duration is reported only for participants with T2DM; therefore, no between-group comparison was performed. A two-sided *p*-value <0.05 was considered statistically significant.

**Table 2:** Vibration perception thresholds at individual anatomical sites of the foot in participants with T2DM and controls.

Anatomical Site	T2DM, Median (IQR), V	Control Group, Median (IQR), V	Unadjusted *p*-Value	Effect Size (r)
Right foot				
Great toe	16.8 (9.5–23.3)	5.3 (3.1–10.7)	<0.001	0.55
1st MTH	16.2 (11.0–24.6)	5.1 (3.0–11.4)	<0.001	0.53
3rd MTH	15.5 (10.1–25.1)	5.2 (2.6–10.6)	<0.001	0.50
5th MTH	14.8 (9.1–25.3)	5.2 (2.2–9.5)	<0.001	0.53
Medial aspect of the midfoot	15.1 (8.5–25.5)	5.0 (2.9–10.1)	<0.001	0.52
Heel	15.6 (9.6–26.6)	4.7 (3.2–11.1)	<0.001	0.54
Central dorsum of the foot	17.4 (10.2–26.8)	6.3 (3.0–11.8)	<0.001	0.48
Medial malleolus	21.3 (12.6–28.6)	6.5 (3.2–14.0)	<0.001	0.54
Left foot				
Great toe	15.7 (10.3–23.1)	4.7 (2.9–10.4)	<0.001	0.55
1st MTH	16.2 (10.1–22.9)	5.2 (2.6–9.8)	<0.001	0.53
3rd MTH	16.0 (9.1–26.8)	6.2 (2.4–10.0)	<0.001	0.54
5th MTH	13.9 (8.9–26.0)	4.9 (2.7–10.6)	<0.001	0.53
Medial aspect of the midfoot	15.3 (9.9–24.9)	4.6 (2.7–10.1)	<0.001	0.55
Heel	15.0 (9.7–26.9)	5.1 (2.8–10.2)	<0.001	0.56
Central dorsum of the foot	15.2 (10.9–25.6)	7.1 (2.7–10.4)	<0.001	0.55
Medial malleolus	19.2 (11.4–27.4)	6.3 (2.9–13.5)	<0.001	0.50

To account for multiple comparisons across 16 tests, a Bonferroni-corrected significance threshold of *p* < 0.003125 (0.05/16) was applied.

**Table 3:** Multivariable linear regression analysis of factors associated with overall VPT.

Variable	B	HC3 SE	β	95% CI for B	*p*-Value
T2DM	6.482	1.514	0.308	3.495–9.477	<0.001
Age	0.263	0.051	0.400	0.162–0.363	<0.001
Sex	0.271	1.448	0.013	−2.588–3.133	0.851
BMI	−0.049	0.149	−0.029	−0.343–0.246	0.743

Dependent variable: overall VPT, calculated as the mean of the integrated VPT values of the right and left feet. R^2^ = 0.367; adjusted R^2^ = 0.351. HC3 heteroskedasticity-consistent robust standard errors were used for coefficient inference.

## Data Availability

The data supporting the findings of this study are available from the corresponding author upon request, subject to applicable ethical and privacy restrictions.

## References

[1] International Diabetes Federation (2025). IDF Diabetes Atlas.

[2] Carmichael J., Fadavi H., Ishibashi F., Shore A.C., Tavakoli M. (2021). Advances in Screening, Early Diagnosis and Accurate Staging of Diabetic Neuropathy. Front. Endocrinol..

[3] Tesfaye S., Brill S., Eerdekens M., Labrador M.M., Petersen G., de Rooij Peek A., Reta A., Ryan D., Schaper N., Tölle T. (2023). Diagnosis, management and impact of painful diabetic peripheral neuropathy: A patient survey in four European countries. J. Diabetes Complicat..

[4] Zhu J., Hu Z., Luo Y., Liu Y., Luo W., Du X., Luo Z., Hu J., Peng S. (2024). Diabetic peripheral neuropathy: Pathogenetic mechanisms and treatment. Front. Endocrinol..

[5] Burgess J., Frank B., Marshall A., Khalil R.S., Ponirakis G., Petropoulos I.N., Cuthbertson D.J., Malik R.A., Alam U. (2021). Early Detection of Diabetic Peripheral Neuropathy: A Focus on Small Nerve Fibres. Diagnostics.

[6] Pop-Busui R., Boulton A.J., Feldman E.L., Bril V., Freeman R., Malik R.A., Sosenko J.M., Ziegler D. (2017). Diabetic Neuropathy: A Position Statement by the American Diabetes Association. Diabetes Care.

[7] Hicks C.W., Selvin E. (2019). Epidemiology of Peripheral Neuropathy and Lower Extremity Disease in Diabetes. Curr. Diab. Rep..

[8] Bodman M.A., Dreyer M.A., Varacallo M.A. (2024). Diabetic Peripheral Neuropathy. StatPearls.

[9] Al-Khafaji M., Otti V. (2025). Early Diagnosis of Diabetic Neuropathy: A Review of Current Diagnostic Tests. Cureus.

[10] Galiero R., Caturano A., Vetrano E., Beccia D., Brin C., Alfano M., Di Salvo J., Epifani R., Piacevole A., Tagliaferri G. (2023). Peripheral Neuropathy in Diabetes Mellitus: Pathogenetic Mechanisms and Diagnostic Options. Int. J. Mol. Sci..

[11] Lauria G., Hsieh S.T., Johansson O., Kennedy W.R., Leger J.M., Mellgren S.I., Nolano M., Merkies I.S., Polydefkis M., Smith A.G. (2010). European Federation of Neurological Societies/Peripheral Nerve Society Guideline on the use of skin biopsy in the diagnosis of small fiber neuropathy. Report of a joint task force of the European Federation of Neurological Societies and the Peripheral Nerve Society. Eur. J. Neurol..

[12] Ribič D., Šarabon N. (2026). Vibration Perception Threshold as a Method for Detecting Diabetic Peripheral Neuropathy: A Systematic Review of Measurement Characteristics. Diagnostics.

[13] Sharma K.N.S., Kumar H.A. (2023). Assessment of the diagnostic accuracy of Vibrasense compared to a biothesiometer and nerve conduction study for screening diabetic peripheral neuropathy. J. Foot Ankle Res..

[14] Mooi C.S., Lee K.W., Yusof Khan A.H.K., Devaraj N.K., Cheong A.T., Hoo F.K., Sulaiman W.A.W., Loh W.C., Jian L.Y., Hui T.X. (2024). Using biothesiometer, Neuropathy Symptom Score, and Neuropathy Disability Score for the early detection of peripheral neuropathy: A cross-sectional study. Qatar Med. J..

[15] Akhtar S., Hassan F., Saqlain S.R., Ali A., Hussain S. (2023). The prevalence of peripheral neuropathy among the patients with diabetes in Pakistan: A systematic review and meta-analysis. Sci. Rep..

[16] Liu M., Gao Y., Chen D.W., Lin S., Wang C., Chen L.H., Ran X.W. (2021). Quantitative vibration perception threshold in assessing diabetic polyneuropathy: Should the cut-off value be adjusted for Chinese individuals with type 2 diabetes?. J. Diabetes Investig..

[17] Hu G., Wu H., Kuang L., Zee B.C., Huang Y., Huang Z., He L., Zeng Y., Gao Y., Wang H. (2021). Clinical study of diabetic peripheral neuropathy screening by retinal vascular geometric parameters. Sci. Rep..

[18] Jadhao P., Swain J., Das S., Mangaraj S., Sravya S.L. (2025). Prevalence and Predictors of Diabetic Peripheral Neuropathy in Newly Diagnosed Type 2 Diabetes Mellitus Patients. Curr. Diabetes Rev..

[19] Mildren R.L., Yip M.C., Lowrey C.R., Harpur C., Brown S.H.M., Bent L.R. (2017). Ageing reduces light touch and vibrotactile sensitivity on the anterior lower leg and foot dorsum. Exp. Gerontol..

[20] Khan N., Ahmad I., Noohu M.M. (2020). Association of disease duration and sensorimotor function in type 2 diabetes mellitus: Beyond diabetic peripheral neuropathy. Somatosens. Mot. Res..

[21] Wiles P.G., Pearce S.M., Rice P.J., Mitchell J.M. (1991). Vibration perception threshold: Influence of age, height, sex, and smoking, and calculation of accurate centile values. Diabet. Med..

[22] Yang Y., Zhao B., Wang Y., Lan H., Liu X., Hu Y., Cao P. (2025). Diabetic neuropathy: Cutting-edge research and future directions. Signal Transduct. Target. Ther..

[23] Coppini D.V., Wellmer A., Weng C., Young P.J., Anand P., Sönksen P.H. (2001). The natural history of diabetic peripheral neuropathy determined by a 12 year prospective study using vibration perception thresholds. J. Clin. Neurosci..

